# Wideband dispersion-free THz waveguide platform

**DOI:** 10.1038/s41598-023-41843-6

**Published:** 2023-09-14

**Authors:** David Rohrbach, Bong Joo Kang, Elnaz Zyaee, Thomas Feurer

**Affiliations:** Institute of Applied Physics, University of Bern, Sidlerstrasse 5, 3012 Bern, Switzerland

**Keywords:** Nonlinear optics, Terahertz optics, Ultrafast photonics

## Abstract

We present a versatile THz waveguide platform for frequencies between 0.1 THz and 1.5 THz, designed to exhibit vacuum-like dispersion and electric as well as magnetic field enhancement. While linear THz spectroscopy benefits from the extended interaction length in combination with moderate losses, nonlinear THz spectroscopy profits from the field enhancement and zero dispersion, with the associated reshaping-free propagation of broadband single- to few-cycle THz pulses. Moreover, the vacuum-like dispersion allows for velocity matching in mixed THz and visible to infrared pump-probe experiments. The platform is based on the motif of a metallic double ridged waveguide. We experimentally characterize essential waveguide properties, for instance, propagation and bending losses, but also demonstrate a junction and an interferometer, essentially because those elements are prerequisites for THz waveform synthesis, and hence, for coherently controlled linear and nonlinear THz interactions.

A variety of THz applications, for instance, communication^1–4^, particle acceleration^5–11^, or spectroscopy^12–15^ and sensing^16,17^ would benefit greatly from a versatile waveguide platform. In the past, a number of different approaches have been proposed^18,19^. Among them different dielectric waveguide geometries showing low attenuation^20–23^ and potentially strong mode confinement^24^. Interestingly, modern three-dimensional printing technologies allow for relatively simple fabrication of integrated dielectric THz waveguide structures^25,26^. Their main drawback is the non-negligible mode dispersion, which severely hampers broadband signal transmission inasmuch as group velocity dispersion leads to strong pulse broadening. While there are various means to compensate for dispersion^27–29^, none of them can be used to transport single cycle THz pulses with octave-spanning frequency bandwidth along the waveguide. Group velocity dispersion is less of an issue in certain metallic waveguides as the majority of the mode propagates in air. For instance, parallel metal plate waveguides show comparable little mode dispersion^30,31^, however, the mode is confined only in one dimension, which limits the achievable field enhancement. Nevertheless, efficient free-space coupling^32–35^ and even fundamental building units of integrated circuits, such as T-junctions^36,37^, were demonstrated. Similarly, metal wire based waveguides exhibit low attenuation and low dispersion^38,39^, but suffer from high bending losses, modest mode confinement, and generally low free space coupling efficiency. Better performance is achieved for two parallel metal wires^40–42^, however, such structures are fragile and often the wires must be encapsulated with a dielectric material to mechanically stabilize the geometry, which in turn increases the mode dispersion and/or the attenuation. A very similar performance is found for slot-line waveguides^43^, which are especially well suited for very compact integrated circuits^44,45^. If no supporting substrate is present, the structure essentially becomes a metallic slit waveguide^46^, which is of special interest since it combines a low group velocity dispersion with a low attenuation and a strong mode confinement^47,48^ across a wide range of frequencies. Slit waveguides consist of two parallel metal ridges separated by an air gap, with the ridge width and the air gap being of similar or smaller size than the free space wavelength. Such structures have been fabricated by milling slits in silicon wafers and by subsequent metal coating^46^, or by carefully aligning two thin metal foils^11^.

Here, we demonstrate a compact and mechanically robust, yet versatile THz waveguide platform essentially using a slit waveguide as the central motif. It features vacuum-like dispersion and moderate propagation and bending losses. Efficient free-space coupling to the fundamental waveguide mode of up to 80% in the frequency range between 0.35 THz and 1 THz is facilitated by a combination of an aspheric lens and a horn antenna. The architecture allows for functional modules and we demonstrate a Y-junction as well as a Mach-Zehnder interferometer in view of coherent THz spectroscopy.

## Methods

We used CST Microwave Studio to optimize the waveguide architecture for strong mode confinement, low dispersion and low losses as well as the coupling antenna for high free-space coupling efficiency in the frequency range of interest between 0.1 THz and 1.5 THz. Experimentally the coupling efficiency and the waveguide properties were disentangled by measuring waveguides with different lengths $L \in \{20\,\textrm{mm}$, $40\,\textrm{mm}$, $80\,\textrm{mm}\}$ using THz time-domain spectroscopy (THz-TDS). Assuming single-mode propagation, the transmitted electric field $E_{\textrm{out}}(\omega )$ and the reference electric field $E_{\textrm{ref}}(\omega )$ (i.e., without the waveguide structure) are related via^30^

1 $$\begin{aligned} E_{\textrm{out}}(\omega ) = E_{\textrm{ref}}(\omega ) \; C_1(\omega ) \; C_2(\omega ) \exp \left[ -\alpha L -\textrm{i}(k_z - k_0) L \right] , \end{aligned}$$

with the angular frequency $\omega$, the coupling coefficients $C_{1,2}(\omega )$, which can be different due to different mode content at the waveguide entrance and exit, the amplitude attenuation coefficient $\alpha$, the *z*-component of the wavevector $k_z$, and the free-space wavevector $k_0 = \omega /c$. A THz near-field imaging setup was used to qualitatively verify the performance of a Y-junction as a prerequisite for a Mach-Zehnder interferometer.

### Numerical simulations

**Figure 1:**
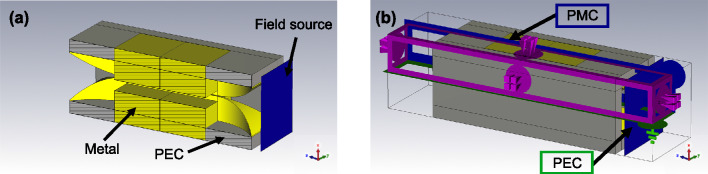
(**a**) Simulation geometry for a 20 mm long waveguide including the in- and out-coupling horn antennas. The focused linearly polarized THz pulse is launched by a near-field source. (**b**) Applied symmetry and boundary conditions, with perfect electrical conductor (PEC) in green, perfect magnetic conductor (PMC) in blue and open boundary conditions in magenta.

**Figure 2:**
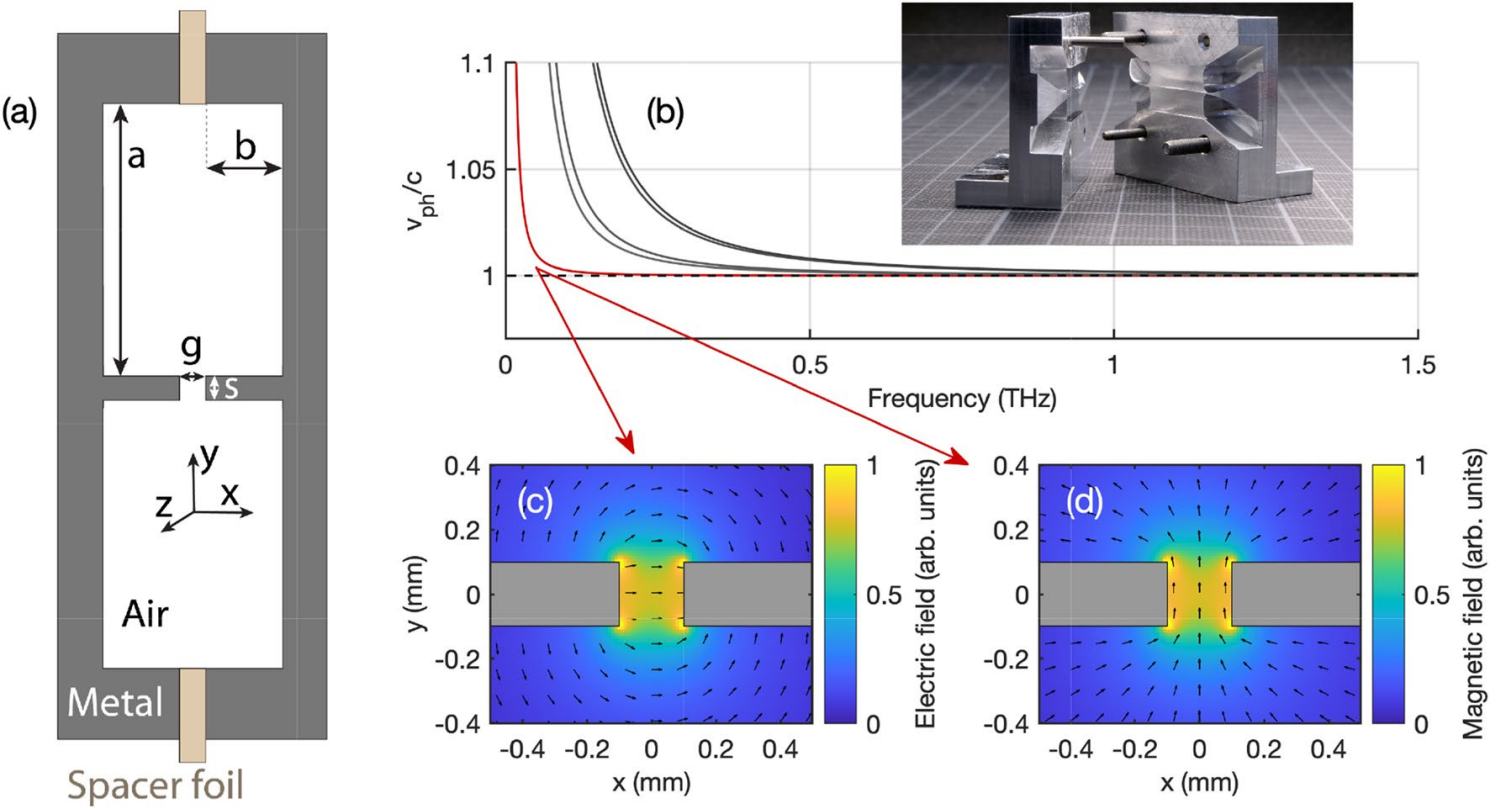
(**a**) Schematic cross-section of the double-ridged waveguide. (**b**) Waveguide dispersion of the lowest five Eigenmodes versus frequency. The inset shows a photograph of the manufactured waveguide structure with a 20 mm long straight channel including in- and out-coupling antennas. (**c**) Electric and (**d**) magnetic field distribution of the fundamental mode in the transverse *xy*-plane at 0.5 THz.

Two types of three-dimensional numerical simulations were performed using CST Microwave Studio. First, we used an Eigenmode solver to determine the first five Eigenmodes together with their dispersive and absorptive properties and their mode profiles, as shown in Fig. 2b–d. We calculated the waveguide modes without any symmetry constraints. Second, we employed a finite difference time domain solver to simulate the entire waveguide, including the horn antennas at both waveguide ends and the converging incident THz pulse as found after the focusing lens. The simulation model is shown in Fig. 1a. The metal (yellow) was aluminum with a THz conductivity of $12\,\hbox {MS}\,\hbox {m}^{-1}$. The top and bottom open boundaries of the horn antennas were set to perfect electric conductor (PEC). The near-field source (at the blue plane) was a converging, broadband THz pulse, after the 50 mm lens, with a temporal shape matched to the measured reference pulse. It was modeled by a Maxwell-Gaussian beam^49,50^ with electric and magnetic field components up to the 5th order correction. To account for the diffraction-limited THz focusing, the beam waist was scaled inversely proportional with frequency with a beam waist of 0.5 mm at 0.6 THz. To reduce the computation time we made use of the symmetry with respect to the *xz*- and *yz*-plane and used perfect magnetic respectively perfect electric boundaries as indicated in Fig. 1b.

### Waveguide design

Figure 2a shows the cross-section of the optimized double-ridged waveguide with the surrounding support structure. It consists of two mirror symmetric metal parts separated by a metallic spacer foil, which defines the gap between the two central ridges. In the limit of $a/s \gg 1$ and $b/g \gg 1$ the geometry resembles essentially that of a slit waveguide with the fundamental mode being confined to the gap volume. For strong mode confinement, both *g* and *s* should in general be similar or smaller than the corresponding THz free-space wavelength, yet keeping in mind that the aspect ratio *b*/*s* is limited by the manufacturing technique. The optimized parameters were found to $b = 1\,\textrm{mm}$, $s = 0.2\,\textrm{mm}$, and $a = 5\,\textrm{mm}$. All waveguide structures except for the THz interferometer were fabricated from aluminum by standard milling machines and subsequent lapping. Alignment holes are used for the accurate assembling of the two mirror symmetric parts. Electric discharge machining was used for the THz interferometer structure with an inner curvature radius reaching 0.2 mm. For this manufacturing technique, we used steel *1.4034* instead of aluminum. The inset in Fig. 2b shows a photograph of the two mirror-symmetric parts for a 20 mm long straight waveguide including the in- and out-coupling horn antennas at both ends of the waveguide. The gap size *g*, i.e. the spacer foil thickness, was varied between $g = 30\,\upmu \textrm{m}$ and $200\,\upmu \textrm{m}$.

The simulated waveguide dispersion relation of the first five Eigenmodes is shown exemplarily for $g = 200\,\upmu \textrm{m}$ in Fig. 2b. The fundamental mode, which resembles a quasi-TEM mode with a cutoff frequency below 10 GHz, exhibits a phase velocity (red curve) very close to the speed of light in vacuum (dashed horizontal line) down to about 0.1 THz. The normalized transverse electric and magnetic field distribution of the fundamental mode are displayed in Figure 2c and d, with color-coded amplitude and arrows indicating the direction of the respective field vectors. While the electric field in the gap area is predominantly polarized along the *x*-direction, the magnetic field is mostly parallel to the *y*-direction. Note that the transverse mode profile is defined by geometry and is approximately frequency-independent. The next four higher-order modes exhibit a mode profile that is not confined to the gap region but to the much larger cross-section of the support structure. The free-space couplers were inspired by the cylindrical tapered couplers used in combination with parallel plate waveguides^35^. Similar geometries were successfully used for local field enhancement^51,52^, but to the best of our knowledge never in combination with a slit waveguide structure. Optimization via numerical simulations resulted in a radius of $13\,\textrm{mm}$ in both transverse dimensions and a total length of 10 mm.

### THz time domain spectrometer

**Figure 3:**
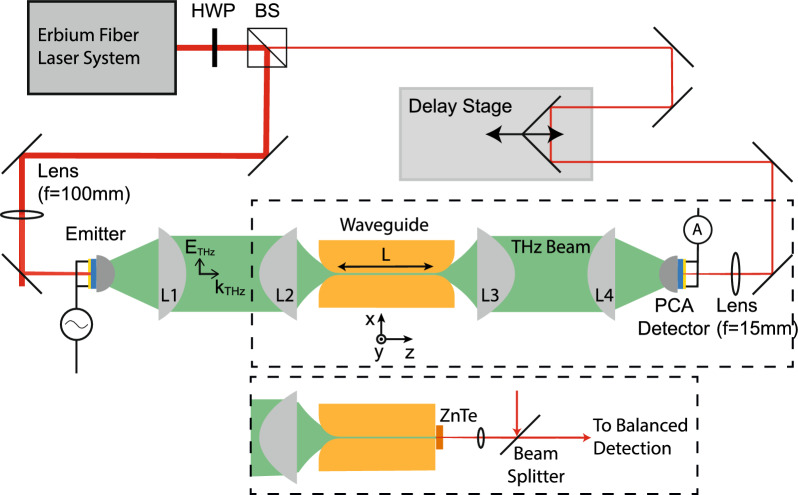
Schematic of the experimental setup: THz pulses are emitted and detected by two PCAs optically gated by a femtosecond laser pulse. The path length of the detector pulse can be adjusted by a delay stage.

A schematic of the THz-TDS is shown in Fig. 3 (top panel). The femtosecond laser pulses come from a *Femto Fiber Pro* by *Toptica*, with a pulse duration of less than 100 fs, a repetition rate of 80 MHz and an average output power of 130 mW at 780 nm. A polarizing beam splitter (BS) sent part of the pulse energy to the emitter and the remaining part to the detector. Approximately 120 mW were used for THz generation at a photoconductive antenna (PCA), which was a broad-area inter-digital PCA *iPCA-21-05-1000-800-h* from *Batop*. About 10 mW were transmitted through the BS and sent to the detector PCA, which was a bow-tie antenna *PCA-100-05-10-800* from *Batop*. The THz-induced current was amplified with a *HF2TA Current Amplifier* from *Zurich Instruments*. For lock-in detection, the bias voltage of the emitter PCA was modulated at 2 kHz and the lock-in integration time constant was set to 1 ms. The emitted THz pulses were imaged from the emitter to the detector by four aspheric high-density polyethylene lenses. The focal length of *L*1 and *L*4 was 100 mm while the focal length of *L*2 and *L*3 was 50 mm. As an input for the simulations, we measured the beam waist at the intermediate focus using the knife-edge method and found a spot size (i.e., beam diameter at 1/$\textrm{e}^2$) of 0.9 mm. To adjust for the different waveguide lengths, the third THz lens *L*3 was mounted on a *z*-axis translation stage.

### THz near-field imaging

THz near-field imaging, shown in Fig. 3 (bottom panel), was used as a qualitative probe to visualize the spatial distribution of the THz radiation at the end of specific waveguide structures. The measurement is very sensitive to the exact distance between the detection crystal and the waveguide exit, hence we did not infer quantitative information from these measurements. Briefly, THz pulses were emitted by a PCA and imaged to the entrance of the waveguide by four off-axis parabolic mirrors. Directly at the exit of the waveguide structures (without horn antenna) the horizontal electric near-field distribution was measured by electro-optic sampling with a $100\,\upmu \textrm{m}$ thick ZnTe crystal. From the opposite direction, a femtosecond laser pulse was focused into the ZnTe crystal and was back-reflected from the interface facing the waveguide. Polarization rotation of the reflected laser beam induced by the THz electric field was subsequently measured using a balanced photo-detection scheme. Spatial imaging was realized by raster-scanning the entire detector unit together with the probe laser beam in *y*-directions relative to the stationary THz beam and waveguide. The spatial resolution was $20\,\upmu \textrm{m}$.

## Results and discussion

### Fundamental mode characterization

**Figure 4:**
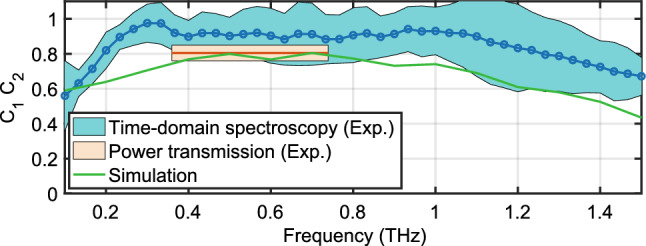
Total coupling coefficient $C_1 C_2$ for the optimal alignment based on time-domain spectroscopy measurements, direct transmission power measurements and numerical simulations.

As stated above, we use waveguides of different lengths to disentangle the coupling efficiency from the waveguide properties. Figure 4 shows the extracted coupling coefficient versus frequency for a gap size of 200 $\upmu$m. The simulations (green curve) predict coupling efficiencies of up to 80% for the frequency range between 0.35 THz and 1 THz and more than 50% between 0.1 THz to 1.4 THz. The simulations indicate that coupling of frequencies below 0.35 THz is reduced by reflection losses at the waveguide entrance due to their larger mode size. Conversely, coupling efficiency for frequencies higher than 1 THz becomes less efficient due to their reduced mode confinement. While the frequency-resolved measurements using THz-TDS (blue open circles) seem to overestimate the coupling (most likely due to imperfect imaging of the reference signal^35^) even though they agree within the error bars (light blue region), the frequency-integrated values using a power meter sensitive to frequencies between 0.35 THz and 0.75 THz agree very well with the measured coupling efficiency.

**Figure 5:**
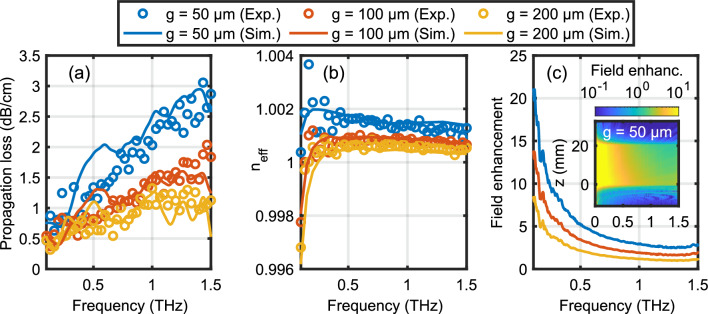
(**a**) Propagation loss, (**b**) effective refractive index, and (**c**) field enhancement at $z = 0\,\textrm{mm}$ as a function of frequency for three different gap sizes of $50\,\upmu \textrm{m}$ (blue), $100\,\upmu \textrm{m}$ (red), and $200\,\upmu \textrm{m}$ (yellow). Open circles correspond to experimental data and solid curves to simulations. The inset in (**c**) shows the simulated color-coded field enhancement as function of frequency and position along the middle of a 20 mm long waveguide with a gap of 50 $\upmu$m.

Figure 5 shows propagation loss, effective refractive index, as well as field enhancement of the fundamental mode as a function of frequency for three different gap sizes of $50\,\upmu \textrm{m}$ (blue), $100\,\upmu \textrm{m}$ (red), and $200\,\upmu \textrm{m}$ (yellow). Generally, we find excellent agreement between measured (open circles) and simulated results (solid curves). The propagation loss increases with frequency, is larger for smaller gap sizes, and is governed mostly by Ohmic losses in the metal (aluminum). With values on the order of a few dB/cm, the propagation power loss is comparable to many other THz waveguides, for instance, microstrip lines, even though there exist exceptions where losses can be even smaller than 0.1 dB/cm in an approximately 0.1 THz wide frequency band^20,23^. Simulations suggest that lower attenuation can be achieved for metals with higher conductivity and should ideally vanish for superconducting materials, but in practice will be limited by scattering at waveguide imperfections. Figure 5b shows that the effective index of refraction deviates at most by about $10^{-3}$ from the vacuum value down to 0.1 THz. The deviation increases as the gap becomes smaller and a similar behavior is found for surface plasmon polaritons on metal wires^53^ or in parallel plate waveguides^54^. The variations of $n_{eff}$ between 0.2 THz to 1.5 THz are less than 0.2%. This exceptional low waveguide dispersion is of utmost relevance for THz-pump optical-probe spectroscopy of gas-phase samples ensuring not only a reshaping-free propagation of single- or few-cycle THz pulses but also a velocity matching between an optical to infrared probe and the THz pulse. The rapid decrease for frequencies below 0.2 THz is related to the fact that the double ridged waveguide has a cut-off frequency of approximately 8 GHz (20 GHz) for a gap size of $30\,\upmu \textrm{m}$ ($200\,\upmu \textrm{m}$). While the onset of this behavior is seen in the dispersion plot, it affects the absorption only closer to the cut-off frequency. The cut-off frequency can be shifted towards lower frequencies, for instance, by increasing the distance *a* between the gap and the sidewalls. We also extract the group velocity dispersion and find $|\beta _2| \le 0.1\,\hbox {ps}\,\hbox {THz}^{-1}\,\hbox {cm}^{-1}$ for frequencies between 0.3 THz to 1.5 THz.

An appreciable electric and magnetic field enhancement — with respect to a free space focused THz pulse — is achieved because the transverse mode profile of the fundamental is smaller than the diffraction-limited free space focus for a THz pulse with the same spectral content. Here, we define the field enhancement factor shown in Fig. 5c in frequency domain as the ratio of the peak electric field amplitude in the waveguide over the peak electric field amplitude at a free space focus using an f# = 1 focusing element. Note that the field enhancement takes into account the coupling efficiency on the input side. The field enhancement can only be inferred from simulations, since we have no means to measure the electric field directly inside the gap. Similar enhancement factors are found for the magnetic field. As for most slit-based structures, lower frequencies show a higher field enhancement. This is because they have a larger mode profile when focused in free space, resulting in a larger increase in electromagnetic energy density when the mode is squeezed in the quasi-TEM mode of the waveguide. The inset in Fig. 5c shows the color-coded field enhancement as a function of frequency and along the propagation axis. Note that especially the low frequency components are already enhanced toward the end of the input coupler ($z < 0$). The field enhancement decreases along the propagation axis in essence due to propagation losses. At 0.5 THz we find a peak field enhancement of 5 and an effective field enhancement of 4.4 when averaged over a 20 mm long waveguide for $g = 50\,\upmu \textrm{m}$. Hence, an intense THz source, for example based on lithium niobate^55–57^, sufficiently strong to produce an electric field strength of 400 kV/cm when focused in free space, can be enhanced to almost 2 MV/cm along the waveguide. Similarly, the corresponding magnetic field would increase from 130 mT to 590 mT.

### Bending, Y-junction and Mach-Zehnder interferometer

**Figure 6:**
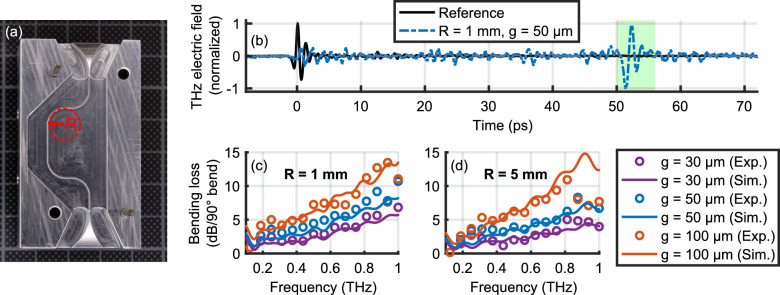
(**a**) Photograph of the bending structure with four 90$^\circ$ bends with an inner radius $R = 5\,\textrm{mm}$. (**b**) Transmitted signal trough a structure with $R = 1\,\textrm{mm}$ and $g = 50\,\upmu \textrm{m}$ together with the normalized air-reference signal (i.e., without waveguide). (**c**) and (**d**) Extracted losses per 90$^\circ$ bend for different gap sizes and bending radii. Circles corresponds to experimental results and solid curves to simulations.

The strong confinement of the fundamental THz mode is especially beneficial for waveguide bends of small radii, on the order of millimeters, leading to low bending loss. Hence, our platform is suitable for developing highly sophisticated THz spectroscopy over a small footprint, for instance, power splitters or micro-ring based filters. In order to characterize the bending loss we use structures as the one shown in Fig. 6a consisting of four 90$^\circ$ bends with radii of $R = 1\,\textrm{mm}$ and $R=5\,\textrm{mm}$. Figure 6b shows the measured transmitted electric field (blue dashed curve) as a function of time for $R = 1\,\textrm{mm}$ and $g = 50\,\upmu \textrm{m}$ together with the normalized reference signal (black solid curve).

The green box indicates the time window in which we expect the signal guided around the four bends to appear. Compared to a straight waveguide the four bends increase the total waveguide length by 15.4 mm corresponding to a 51 ps time delay. The smaller signal contributions before and after the main guided signal are either due to scattering or leaking out of the waveguide. For example, the signal around 0 ps corresponds to a direct line-of-sight leakage through the structure. To extract only the guided signal we applied a Tukey time-window^58^ (with cosine fraction of 0.5) centered at 54.5 ps with a length of 15 ps. Note that the simulations are based on a single 90$^\circ$ bend to clearly separate the guided signal from other contributions. Figure 6c and d show the losses per 90$^\circ$ bend as a function of frequency, for different gap sizes and bending radii. Generally, we find excellent agreement between experimental (open circles) and simulated results (solid curves). There is essentially no difference in performance when decreasing the radius from 5 mm to 1 mm. Even though a bending radius of $R=1\,\textrm{mm}$ is smaller than the free-space wavelength for frequencies below 0.3 THz, we observe efficient guiding. Experiments as well as simulations show that the bending losses are approximately proportional to $g \omega$, which suggests that the underlying loss mechanism is the mode mismatch between the guided and the free-space mode. For gaps larger than 100 $\upmu$m no signal guiding was observed, but for smaller gaps guiding improves irrespective of frequency. Hence, there is a trade-off between bending and propagation loss, which can be optimized by adjusting the gap size.

**Figure 7:**
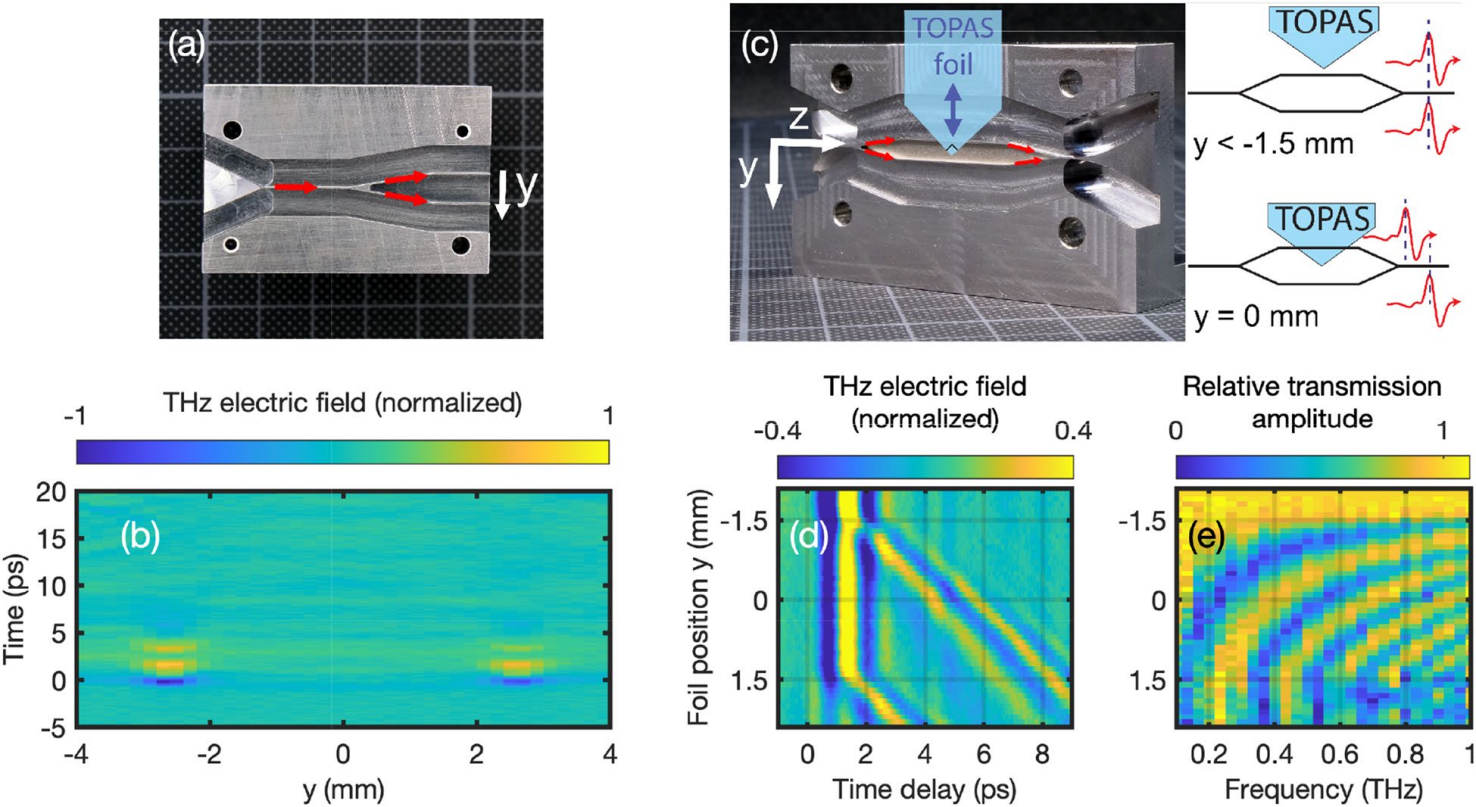
(**a**) Photograph of the Y-junction with in-coupler. (**b**) Measured electric field as a function of *y*-position and time. (**c**) Photograph of Mach-Zehnder interferometer based on two identical Y-junctions with in- and out-coupler. Position and geometry of the TOPAS foil are indicated by the blue shape. (**d**) THz electric field at the output of the interferometer for different foil position and (**e**) corresponding spectra.

THz waveform synthesizers for coherent control of linear and nonlinear interactions with matter are often realized via spectral phase and/or amplitude shaping. Integrated versions of such devices require as fundamental building blocks power splitters and there are a number of different options ranging from a standard Y-junction to a $1 \times 2$ rib-directional coupler, a parabolic-shaped structures, or a $1 \times 2$ arrow-2D directional coupler to name but a few^59^. Here, we fabricated a Y-junction and based on that a Mach-Zehnder interferometer as a simple but fundamental waveform synthesizer. Various other designs for THz Mach-Zehnder interferometers have already been demonstrated^60,61^. However, none of them is based on zero dispersion waveguide structure. A photograph of the Y-junction is shown in Fig. 7a. The branching angle is kept as low as $20^\circ$ to minimize losses and the distance between the two parallel output channels is 5.2 mm. Figure 7b shows the color-coded THz electric field as a function of time and *y*-position measured at the edge of the Y-junction structure using the near-field detection unit. Indeed, we find that the THz pulse is split in two replica with similar amplitude and time dependence. Small deviations from the designed symmetric splitting ratio of 50:50 are probably due to minor defects at the channel ends leading to different detection efficiencies. Note that the splitting ratio can be tuned by adjusting the branching angles of the two channels.

A photograph of the THz interferometer structure is shown in Fig. 7c. Basically, it consists of two mirror symmetric Y-junctions with two parallel waveguide sections in between. The separation of the two parallel waveguide sections is 3 mm. In order to delay the replica in the upper interferometer arm with respect to the other, we mount a 90$^\circ$-tip made of a 80 $\upmu$m thick dielectric foil on a linear stage moving the foil in *y*-direction in and out of the 100 $\upmu$m gap. The further the foil is moved in the gap, the larger the delay. The selected foil material, the cyclic olefin copolymer TOPAS, ensures low THz absorption^62^. Figure 7d shows the color-coded THz electric field after the interferometer as a function of time and foil position. The foil was moved in steps of 100 $\upmu$m for a total distance of 4.5 mm. At position -2 mm the foil is ineffective and the two replica superimpose without any delay between them. For positions larger than -1.5 mm one replica is delayed with respect to the other and we find a maximum delay of about 6 ps at 1.5 mm. For positions larger than 1.5 mm also the second waveguide section is affected by the TOPAS foil such that both replicas are delayed symmetrically. Note that a fraction of the pulse is not guided by the waveguide structure but propagates at the direct line-of-sight and is therefore mostly unaffected by the TOPAS foil. As discussed for the bending structure, this leakage signal could be reduced by further reducing the gap size. Also note that the delayed signal is slightly less intense due to Fresnel reflections and absorption. In addition, we observe an increasing dispersion as we move the TOPAS foil further in and simulations suggest this effect to be due to the air-gap between the TOPAS foil and the metal structure^63^. Figure 7e shows the normalized color-coded THz spectra versus frequency and foil position and we observe the typical interference pattern for two time delayed THz pulses with the spectral fringes becoming more dense as the time delay increases. Well-controlled time delays between two or three phase-coherent THz pulses are essential for 2D-THz spectroscopy. Here for instance, the interferometer output can be coupled to a waveguide containing the sample and the nonlinear signal exiting the waveguide is sent to an electro-optic crystal for detection. For a three THz pulse interaction, the interferometer can be easily modified to produce an adjustable three pulse sequence.

## Conclusion

We have demonstrated a versatile THz waveguide platform featuring broadband performance, bending and propagation losses of a few dB/cm, vacuum-like dispersion, efficient free-space coupling, and field enhancement factors of up to 5. Above 0.1 THz its performance is essentially frequency independent such that single- or few-cycle THz pulses, like those produced by optical rectification, propagate along the waveguide without reshaping. The extended interaction length combined with the field enhancement boost any nonlinear THz light matter interaction by orders of magnitudes. Moreover, the close to one effective refractive index of the fundamental mode allows for velocity matching between a THz pump and a visible to infrared probe pulse and hence for THz-pump optical-probe spectroscopy over the entire waveguide length. In addition, we have demonstrated a power-splitter and based on that an interferometer for THz waveform synthesis. Hence, this platform can be used to construct a compact spectroscopy system for coherently controlled linear or nonlinear spectroscopy.

## Data Availability

Data (including CAD-files of the waveguides) underlying the results presented in this paper are available in the BORIS repository, Ref.^64^.
